# Superficial esophageal squamous cell carcinoma with esophageal lichen planus and esophageal epidermoid metaplasia treated with endoscopic submucosal dissection

**DOI:** 10.1007/s12328-025-02190-3

**Published:** 2025-07-28

**Authors:** Naoya Masuda, Kenji Yamazaki, Ryoji Kushima, Daiki Hirota, Hiroki Taniguchi, Noritaka Ozawa, Shogo Shimizu, Masahito Shimizu

**Affiliations:** 1 Department of Gastroenterology, Gifu Prefectural General Medical Center, 4-6-1 Noishiki, Gifu, 500-8717 Japan; 2 Department of Gastroenterology, Gifu University School of Medicine, 1-1 Yanagido, Gifu, 501-1194 Japan; 3 Department of Pathology, Shiga University of Medical Science, Seta Tsukinowa-Cho, Otsu, Shiga 520-2192 Japan

**Keywords:** Esophageal lichen planus, Esophageal epidermoid metaplasia, Esophageal squamous cell carcinoma, Endoscopic submucosal dissection, Esophagus

## Abstract

We present a case of a 70-year-old female patient who was initially diagnosed with oral lichen planus at a dental and oral surgery clinic. She underwent an upper gastrointestinal endoscopy for further evaluation of her dysphagia. Endoscopic and histopathological examinations revealed that she had superficial esophageal squamous cell carcinoma, coexisting with a background of esophageal lichen planus and esophageal epidermoid metaplasia. She underwent endoscopic submucosal dissection, recovering with minimal stenosis symptoms. Recent reports suggest that esophageal involvement associated with lichen planus is often unrecognized or misdiagnosed. Another esophageal disease with a similar chronic inflammatory background is esophageal epidermoid metaplasia. The coexistence of esophageal squamous cell carcinoma with esophageal lichen planus and esophageal epidermoid metaplasia have been reported; however, few studies with detailed endoscopic and pathologic findings are available. We present an interesting case of squamous cell carcinoma against a background of esophageal lichen planus and esophageal epidermoid metaplasia, while also considering the carcinogenic processes involved.

## Introduction

Esophageal lichen planus (ELP) is most frequently observed in middle-aged women [1] and often presents with symptoms of dysphagia and odynophagia [2]. Endoscopy is necessary for the diagnosis of ELP; however, not all patients with lichen planus (LP) undergo this procedure, raising concern that ELP may be underdiagnosed [3]. Since ELP develops against a background of chronic inflammation, reports of carcinogenesis have been documented; however, the number of cases remains small, and no standardized observation or treatment protocols have been established.

Another esophageal disease with a similar chronic inflammatory background is esophageal epidermoid metaplasia (EEM). EEM is characterized by a stratified squamous epithelium with a thick stratum corneum and a granular layer with keratohyalin granules beneath the stratum corneum [4]. Reports of ELP coexisting with EEM are considerably rare [5]. Although some studies have suggested an association between EEM and esophageal squamous cell carcinoma (ESCC), the relationship remains unclear.

In this report, we describe a rare case of superficial ESCC coexisting with EEM in the background of ELP, with a literature review on the diagnosis, treatment strategy, and course of the disease.

## Case report

**Fig. 1:**
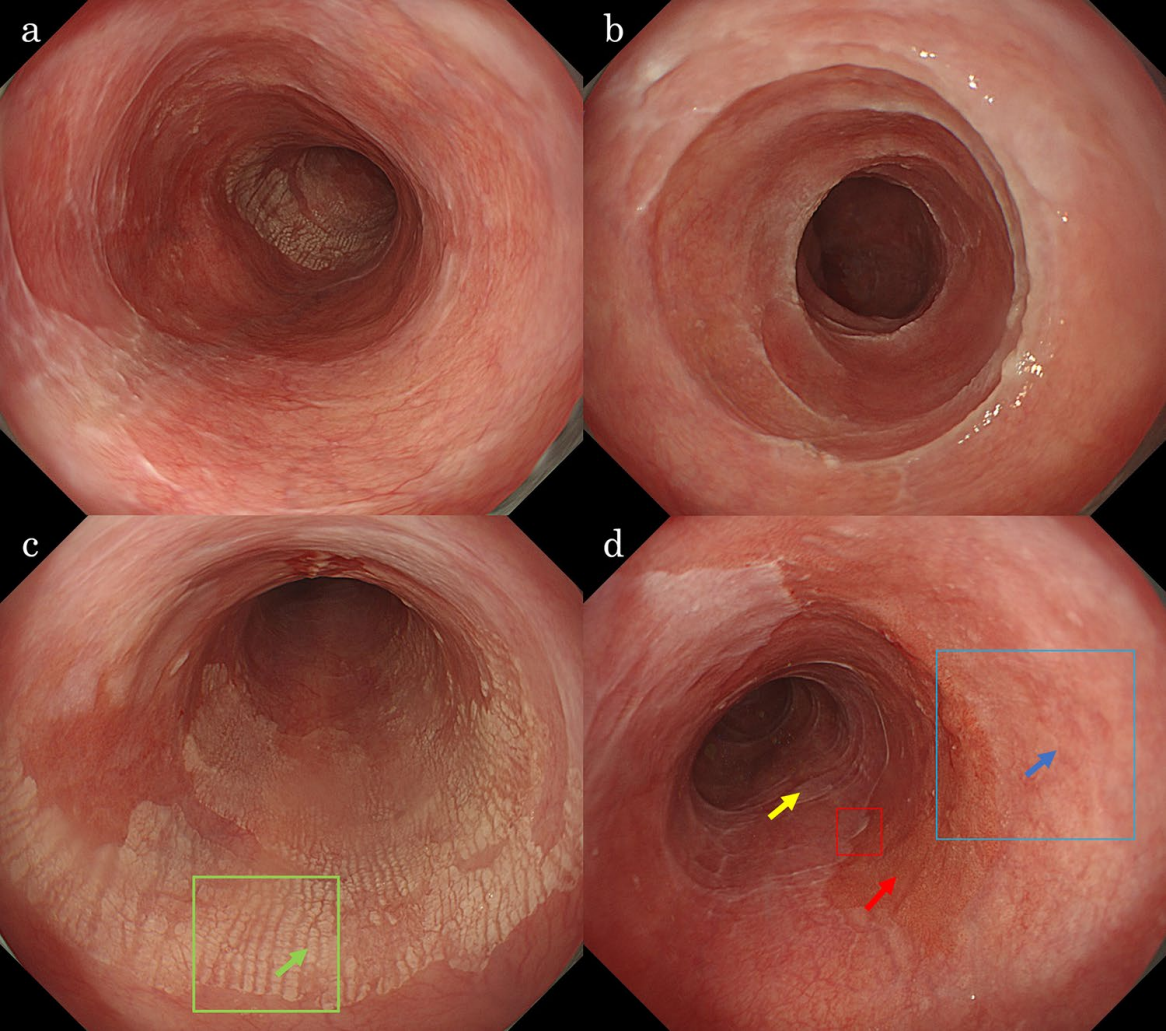
Endoscopy findings (white light). The finding of whitish-lacy mucosa (**a**). Trachea-like changes (**b**). White squamous ridges. The ridges are aggregated regularly and are covered with a thick epithelium (**c**, 25 cm from the incisor). Erythematous depression area, absent during the initial examination, is observed. A pale erythematous area extends to the oral side of the erythematous depression area (**d**, 28 cm from the incisor)

**Fig. 2:**
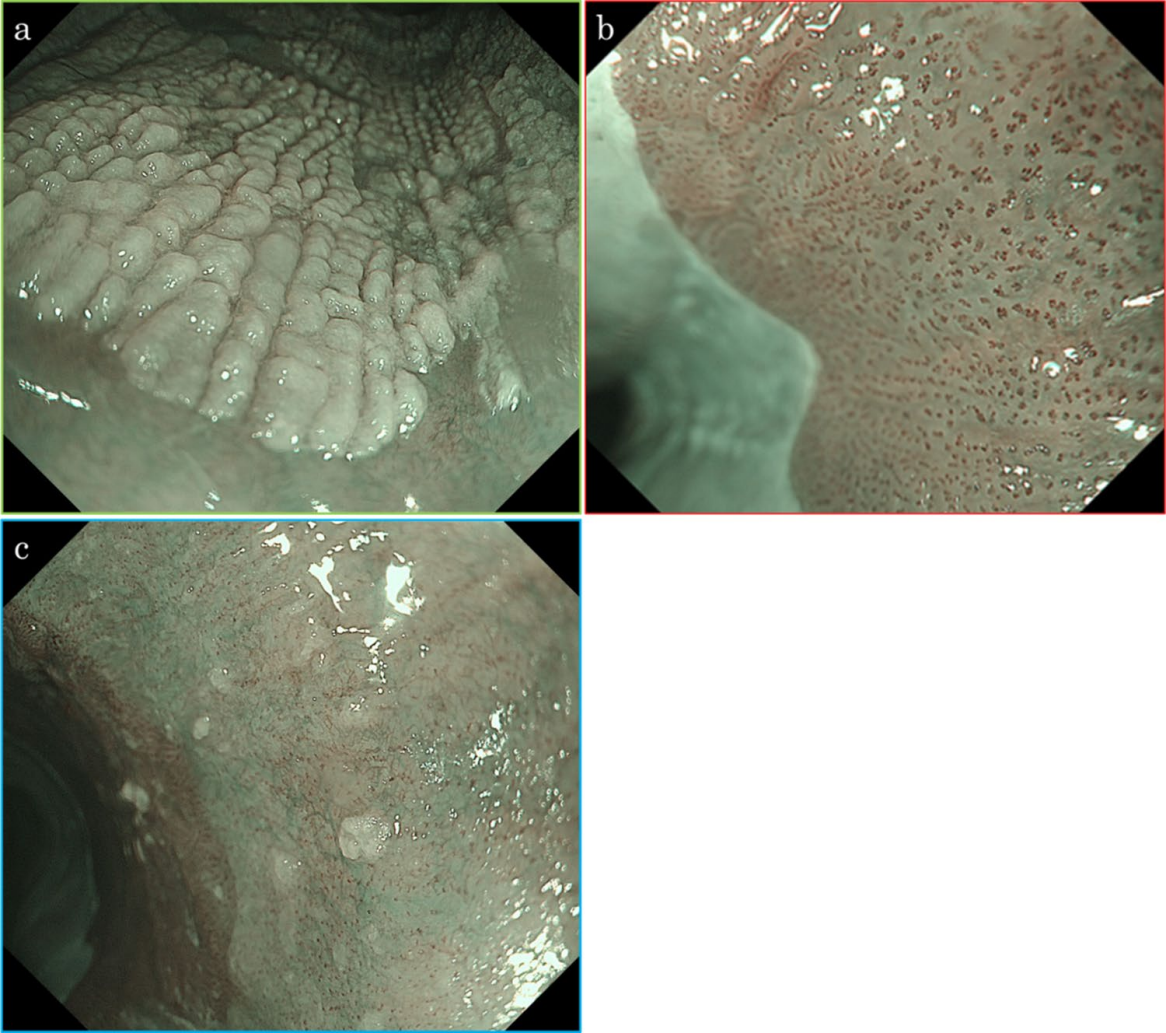
Endoscopy findings (narrow band imaging [NBI]). The white squamous scaly ridged area (a, green frame in Fig. 1c) showed no blood vessels visible. The erythematous depression area (b, red frame in Fig. 1d) showed dot-shaped blood vessels with dilated, meandering, and uneven caliber and shape are observed. The oral side of erythematous depression area (c, blue frame in Fig. 1d) showed slight vascular hyperplasia with minimal variation in caliber and no heterogeneity in shape is observed

**Fig. 3:**
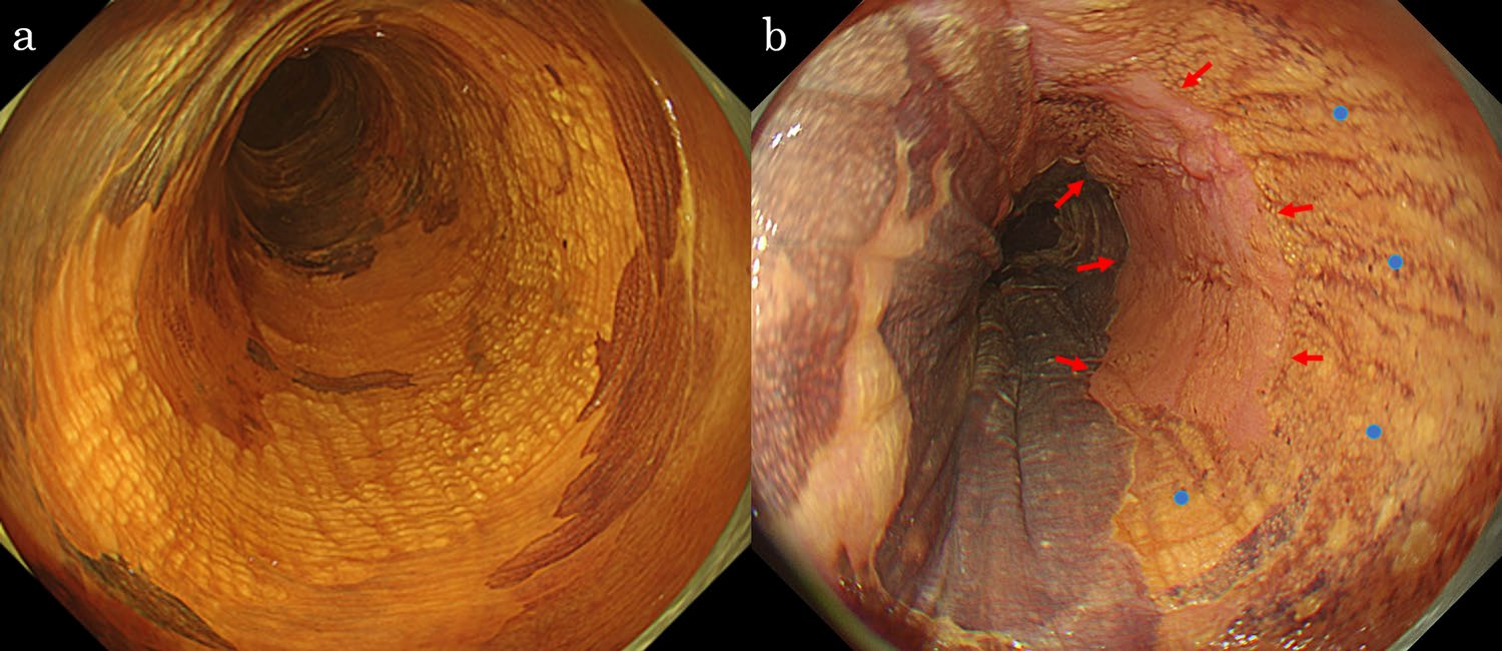
Endoscopy findings (iodine staining). The white squamous ridge is iodine-negative. The surrounding whitish-lacy mucosa exhibits various iodine staining, ranging from light to negative (**a**). The erythematous depression shows iodine-negative and is positive for the PC sign (**b**, red arrow). The oral side of this, the pale erythematous area is negative for PC signs and shows light iodine staining (**b**, blue circle)

A woman in her 70 s was diagnosed with oral LP at a dental and oral surgery clinic and underwent follow-up observations. She had a medical history of hypertension and was administered oral spironolactone and amlodipine. She reported consuming approximately 20 g of alcohol daily and had a history of smoking, which she quit 40 years ago. She presented with complaints of chest discomfort and difficulty swallowing. Physical examinations of the chest and abdomen revealed no significant abnormalities; therefore, an upper gastrointestinal endoscopy was performed for a detailed examination. The results showed whitish-lacy mucosa and aggregated white squamous ridges in the thoracic esophagus. No evidence of gastroesophageal reflux disease or Barrett’s esophagus was found. Histologic examination of biopsied specimens confirmed a diagnosis of ELP for the whitish-lacy mucosa and EEM for the white squamous ridges. The patient was administered proton pump inhibitors and oral budesonide; however, the symptoms did not improve. The symptoms were mild and the patient underwent follow-up observations. Two years after the initial examination, she underwent an upper gastrointestinal endoscopy procedure as a follow-up observation. The entire esophagus was slightly narrowed, exhibiting increased resistance to endoscopic passage. The esophageal mucosa exhibited whitish-lacy mucosa that easily exfoliated (Fig. 1a), and trachea-like changes (Fig. 1b). White squamous ridges presented with a fluffy, scaly surface (Fig. 1c). Compared to the initial examination, both the whitish-lacy mucosa and the white squamous ridges had significantly increased in size. An erythematous depression, extending distally from the white squamous ridged lesion, was observed (Fig. 1d). Narrow-band imaging (NBI) magnification revealed no visible blood vessels within the white squamous ridged area (Fig. 2a). In contrast, the erythematous depression exhibited dot-shaped blood vessels, consistent with Japan Esophageal Society classification type B1 (Fig. 2b) [6]. An area of subtle mucosal irregularity was also observed oral to the erythematous depression, showing increased vascularity (Fig. 2c). The white squamous ridged area was iodine-negative. The whitish-lacy mucosa exhibited variable iodine staining, ranging from light to negative (Fig. 3a). The erythematous depression was iodine-negative in chromoendoscopy with iodine staining and positive for the pink color (PC) sign, characterized by a dramatic color change after iodine staining, transitioning from the initial yellow color to pink within 2–3 min (Fig. 3b) [7].

**Fig. 4:**
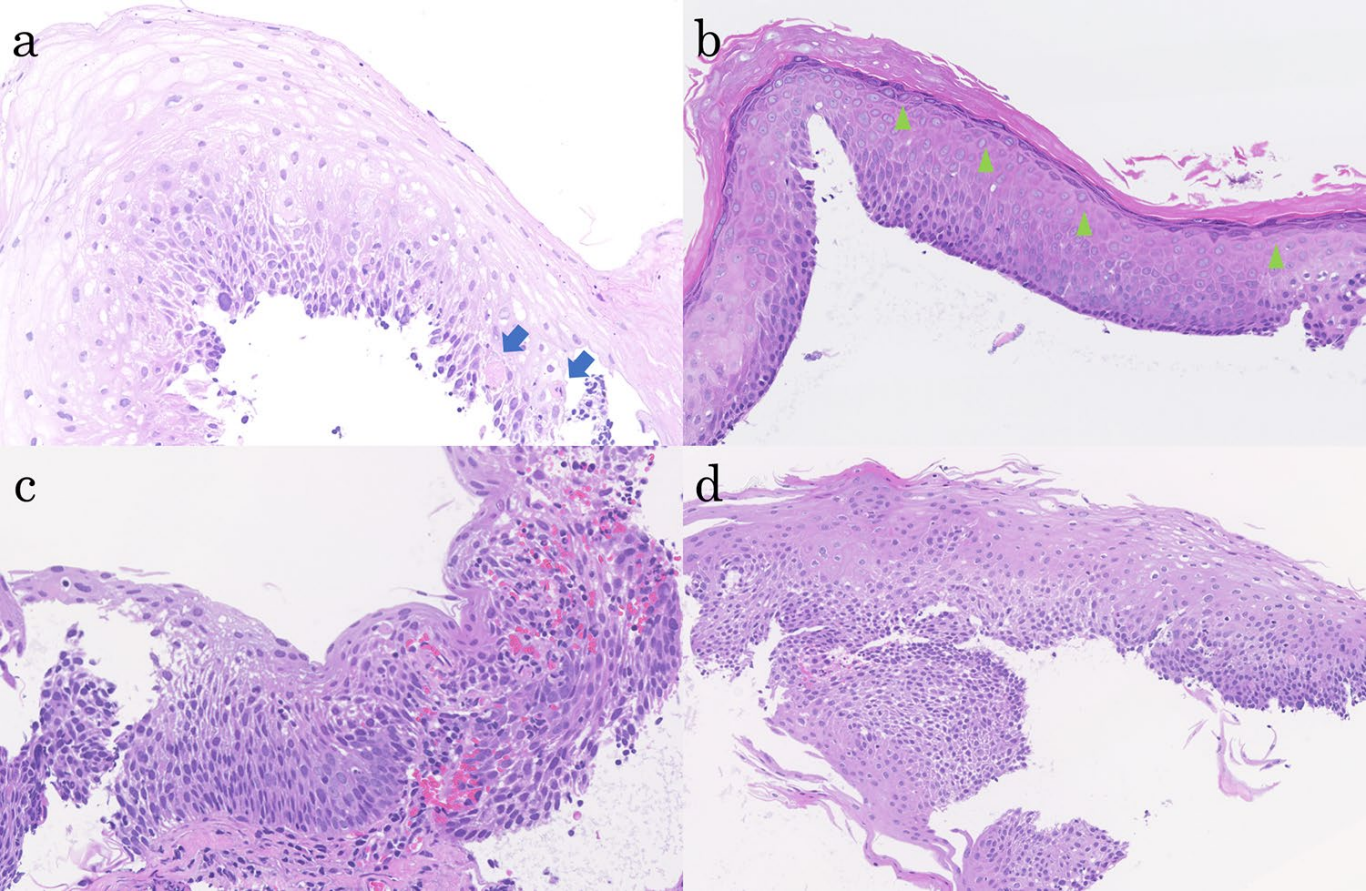
Histopathological findings in biopsy. Histopathological findings from biopsies of the whitish-lacy mucosa (**a**, yellow arrow in Fig. 1d). The squamous epithelium is irregularly thickened, and the basal layer is disorganized. Civatte bodies are seen in the lower epithelial layer with mild lymphocytic infiltration (**a**, blue arrows). Nuclear enlargement and disorganization are observed, but the atypical cells are not regional, and superficial differentiation is clear (hematoxylin–eosin staining, X40). Histopathological findings from biopsies of the white squamous ridges (**b**, green arrow in Fig. 1c). The stratified squamous epithelium has a thick keratinized layer on its surface, with a granular layer containing keratohyalin granules directly below it (b, green arrowhead) (hematoxylin–eosin staining, X40). Histopathological findings from biopsies of the erythematous depression (c, red arrow in Fig. 1d). Atypical cells with nuclear atypia, including spindle-shaped cells, were observed in almost all layers except for the outermost layer, with observations equivalent to SCC (hematoxylin–eosin staining, X40). The light iodine staining area on the oral side of the erythematous depression (**d**, blue arrow in Fig. 1d). Superficial differentiation was observed, and atypical cells with a disturbed nuclear arrangement and nuclear atypia were observed near the middle layer of the epithelium, with observations equivalent to SIN (hematoxylin–eosin staining, X40)

Histologic examination of biopsied specimens confirmed a diagnosis of ELP for the whitish-lacy mucosa (Fig. 4a) and EEM for the white squamous ridges (Fig. 4b). A biopsy from the erythematous depression, which was positive for the PC sign, showed features consistent with SCC according to the Japanese classification criteria (corresponding to high-grade dysplasia [HGD] according to the World Health Organization [WHO] criteria) (Fig. 4c). Similarly, a biopsy of the lightly iodine-stained area surrounding the erythematous depression, which was PC sign negative, revealed squamous intraepithelial neoplasia (SIN) according to the Japanese classification (corresponding to low-grade dysplasia [LGD] according to the WHO criteria) (Fig. 4d).

The patient was diagnosed with superficial ESCC with concomitant EEM on a background of ELP. Preoperative fluorodeoxyglucose positron emission tomography/computed tomography revealed no evidence of local lymph node or distant metastasis.

Given the potential for ESCC development in EEM areas [8–10], a circumferential lesion measuring 6 cm in length, including the SIN and EEM, was resected via endoscopic submucosal dissection (ESD). Informed consent was obtained regarding the risk of postoperative stenosis and the need for post-ESD steroid therapy to prevent stricture formation.

The final pathologic diagnosis was ESCC, SIN, EEM, and ELP. The pathologic T stage was stage 1a with invasion limited to the lamina propria mucosa (pT1a-LPM). No lymphatic invasion (Ly0) or vascular invasion (V0) was observed. Both the pathologic horizontal (pHM0) and vertical margins (pVM0) were negative.

**Fig. 5:**
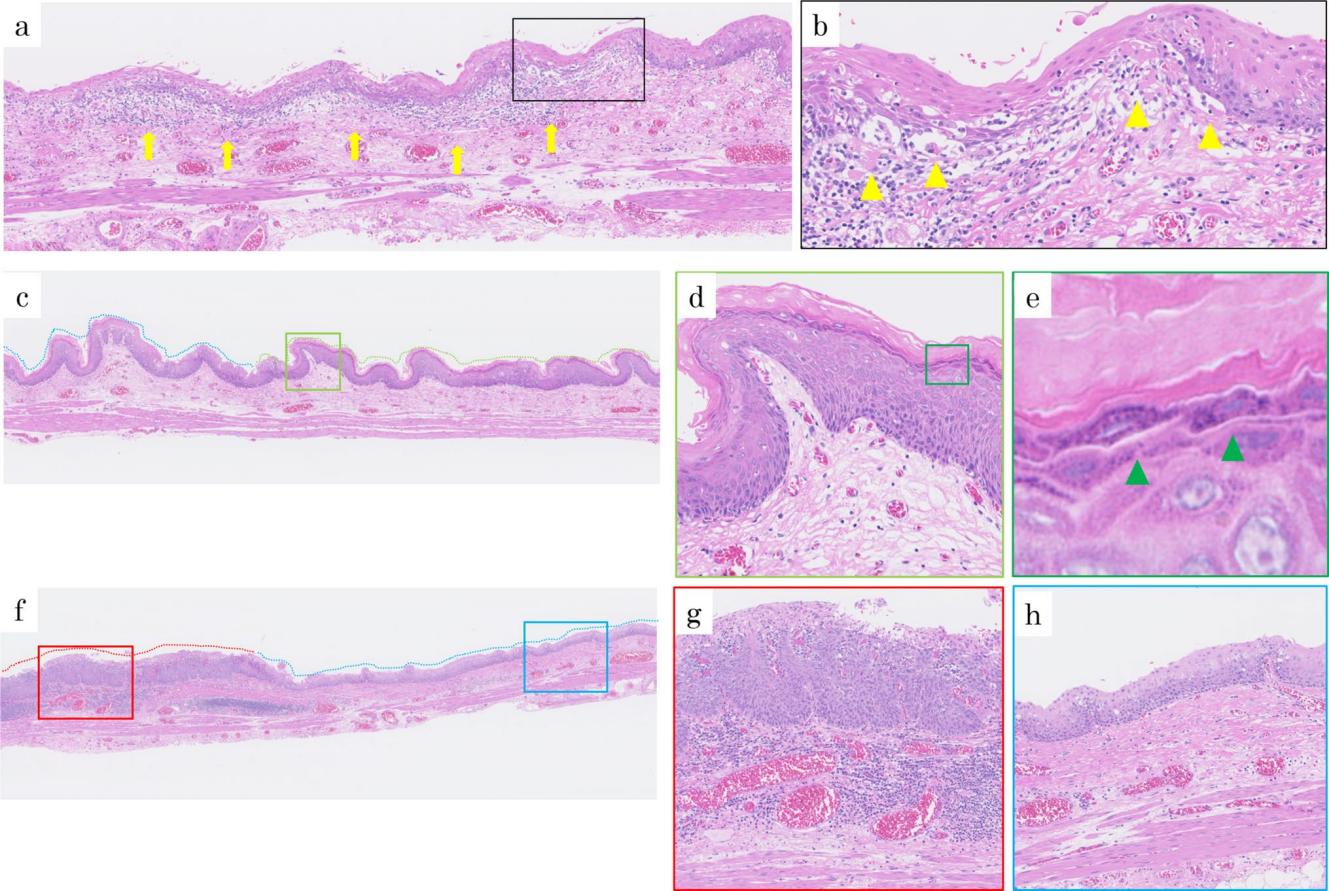
Histopathological findings of the resected specimen. LP findings **a**, **b** Band-shaped inflammatory cell infiltration (**a**, yellow arrow), and Civatte bodies (**b**, yellow arrowhead [black frame in **a**]) are observed between the squamous epithelium and lamina propria. EEM findings (**c**, **d** [light green frame in **c**], **e** [green frame in **d**]; green dotted line in **c**): A prominent keratinized layer is observed on the surface layer (**d**), and a granular layer containing keratohyalin granules is identified immediately below (**e**, green arrowhead) (hematoxylin–eosin staining, **c** X8, **d** X100, **e** X200). SCC findings (**f**, **g** [red frame in **f**]: red dotted line in **f**): The erythematous depression (red dotted line in **f**) represents SCC. Atypical cells with disorganized nuclear arrangement and nuclear atypia, including spindle-shaped cells, are observed in almost all layers except for the outermost layer. SIN findings (f, h [blue frame in **f**]; blue dotted line in **c**, **f**): The oral side of the erythematous depression (blue dotted line in **c**, **f**) represents SIN. Atypical cells with disorganized nuclear arrangement and nuclear atypia were observed up to the middle layer of the epithelium. (hematoxylin–eosin staining, **f** X8, **g** X200, **h** X200)

The background mucosa exhibited band-like inflammatory cell infiltration that was characteristic of LP. Civatte bodies were also observed (Fig. 5a, b). A prominent keratinized layer was noted on the surface layer of the white squamous ridged area, and a granular layer containing keratohyalin granules could be clearly seen just below it (Fig. 5c, d, e). The erythematous depression area findings were consistent with SCC (Fig. 5f, g), and the lightly iodine-stained area surrounding the erythematous depression was diagnosed as SIN (Fig. 5f, h).

**Fig. 6:**
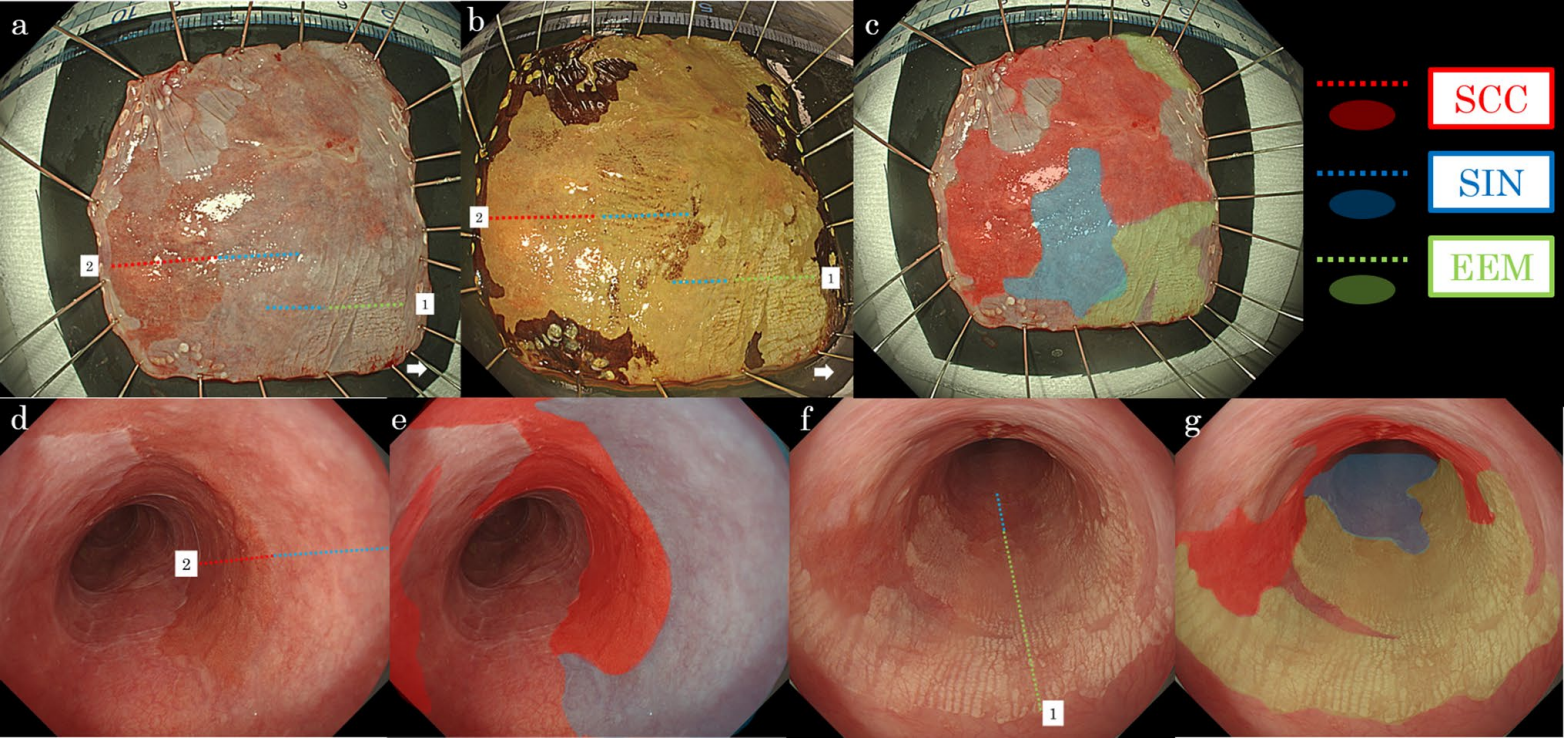
Comparison. Comparison between histopathological findings and freshly resected specimens (**a**–**c**) and a comparison between histopathological findings and endoscopic images are presented (**d**–**g**). The dotted line No. 1 (in **a**, **b**, **f**) shows the location of the pathologic sections presented in Fig. 5c. The dotted line No. 2 (in **a**, **b**, **d**) shows the location of the pathologic sections presented in Fig. 5f. Red areas and dotted lines indicate SCC, blue areas and dotted lines indicate SIN, and green areas and dotted lines indicate EEM

The comparison between histopathological findings and freshly resected specimens (Fig. 6a–c) and between histopathological findings and endoscopic images are presented (Fig. 6d–g). In addition, the lesion encompassing areas of SCC exhibited a subcircumferential spread.

On the day of the ESD procedure, triamcinolone (150 mg total dose) was injected into the submucosal layer, and prednisolone (30 mg/day) was administered orally. The prednisolone dose was reduced by 5 mg every 3 weeks. The postoperative course was smooth; however, 18 weeks post-ESD, the patient reported mild chest discomfort, and endoscopy showed slight esophageal stenosis. Thus, endoscopic balloon dilation and local steroid injection were performed. Nine months post-ESD, the patient has shown almost no symptoms of stenosis.

## Discussion

Typical ELP endoscopic findings include pale, edematous thickened mucosa, whitish-lacy mucosa, and loss of the normal vascular structure [3]. These findings can newly emerge during endoscopy, as mucosa is easily dislodged by scope contact [11, 12]. Endoscopists should recognize these findings as indicative of ELP. Endoscopic findings of ELP typically involve the upper to middle esophagus, unlike acid reflux disease [3]. Histopathologically, ELP is characterized by a dense band-like subepithelial lymphocytic infiltrate, luminal exudate, basal layer degeneration, and Civatte bodies [1, 2, 13].

EEM is another esophageal disease associated with chronic inflammation. It is characterized by a stratified squamous epithelium with a thick stratum corneum overlying a granular layer containing keratohyalin granules [4]. Endoscopically, EEM appears as slightly raised, white, flattened ridges with sharp borders and a shaggy, scaly surface. Iodine staining is typically negative [4]. NBI magnification reveals a thick keratinized layer covering the lesion surface, with no visible blood vessels [4]. Background diseases that cause EEM include gastroesophageal reflux disease, Barrett’s esophagus, and ELP. In a report of 40 cases of EEM, 32 (80%) had gastroesophageal reflux disease, seven (18%) had Barrett’s esophagus, and five (13%) had ELP. When EEM is detected in the proximal esophagus, the possibility of underlying ELP should be considered, as demonstrated in our case [5].

Recognizing the increased malignancy risk in patients with ELP is crucial. While the initial trigger of ELP remains unclear, an autoimmune mechanism is likely involved [14]. ELP is a chronic inflammatory condition marked by cytotoxic CD8 + T cells inducing apoptosis in basal keratinocytes, alongside regulatory and helper T cells [15–17]. Chronic inflammation raises concerns about malignant transformation, as documented in case reports [18, 19]. Ravi et al. found cancer in eight (6.1%) of 132 ELP cases [20]. In addition, chronic stimulation, such as curettage, has been linked to a higher risk of SCC in LP [19]. This suggests that chronic stimuli such as smoking and alcohol abuse may also heighten ESCC risk in ELP. In this case, the patient had a history of drinking and smoking.

EEM is often found near ESCC [8]. A study using targeted next-generation sequencing revealed that 12 of 18 (67%) EEM specimens had gene alterations (TP53, PIK3CA, EGFR, MYCN) associated with ESCC [9]. Armit et al. reported ESCC in nine (23%) of 40 EEM cases [5]. EEM should be recognized as a preneoplastic lesion [8] and monitored carefully for ESCC. Endoscopically, the thick stratum corneum of EEM obscures vascular structures in NBI magnification. Thus, evaluating cancer complications only using endoscopic findings proves difficult. In this case, multiple endoscopic examinations and biopsies were required to assess lesion extent.

Stenosis can occur in ELP due to chronic inflammation. Endoscopic balloon dilatation is a common procedure; however, the dilating stimulus can induce the formation of new LP lesions [21, 22]. Therefore, when performing endoscopic treatment, careful attention must be given to stenosis post-treatment. Local or systemic steroids are recommended for endoscopic treatment of ESCC with more than three-fourths circumferential involvement to prevent post-treatment strictures [23]. In this case, the resected lesion was circumferential and approximately 6 cm in length, raising concerns about potential stenosis after ESD. However, after local triamcinolone injection and systemic prednisolone administration, no significant stenosis symptoms were noted 9 months after ESD.

In conclusion, we have described a case of superficial ESCC combined with EEM on a background of ELP. ESD is an option for the treatment of ESCC associated with ELP with an appropriate treatment to prevent stenosis.

## Abbreviations

ELP: Esophageal lichen planus
EEM: Esophageal epidermoid metaplasia
SCC: Squamous cell carcinoma
ESCC: Esophageal squamous cell carcinoma
NBI: Narrow band imaging
PC: Pink color
HGD: High-grade dysplasia
SIN: Squamous intraepithelial neoplasia
LGD: Low-grade dysplasia
ESD: Endoscopic submucosal dissection
